# The Juggling of Statistics

**Published:** 1905-11

**Authors:** 


### The Juggling of Statistics.

WHEN Dr. Stokes of the navy read his paper at the meeting of the Military Surgeons' Association at Detroit in September, attacking Dr. Louis L. Seaman's figures of mortality during the Spanish war, he unconsciously, if not quite picturesquely, performed a great service which will ultimately be of benefit to the medical department of the United States army. Dr. Seaman spoke of Japan's conquest of "the silent foe"—disease—and showed that contrary to our experience in the Spanish war the Japanese casualties from wounds were many times larger than the deaths from disease. Dr. Stokes criticised Dr. Seaman's American figures and said there were 240 deaths from wounds and 400 from disease. Further he severely condemned, with a note of bitterness, Dr. Seaman's incorrectness as to figures.

Plainly, there was jugglery of statistics here. Either Dr. Seaman had, for some unknown reason, made his figures showing deaths from disease during the Spanish war larger than they should have been and than they actually were, or else Dr.

Stokes, with official records at hand, had overlooked some of the returns. The JOURNAL believes that the medical department of the United States army is numerically too small to be successful; that its officers have insufficient rank and far too little power. The medical department is composed of able men; but the system under which they work is, to say the least, distinctly bad. It has been designated as "rotten" by men who have fallen under its pernicious influence. It is the same system which held up medical supplies at Tampa that mules might be forwarded to Cuba; it is the same system which made it possible to so insult the medical profession that physicians under contract, en route to the Philippines were quartered in the holds of transports while staff officers' friends occupied staterooms and suites; the system which stripped the acting assistant surgeon of his title and uniform and made him a "contract" surgeon, labeled and unable to give an order which even the enlisted man of the hospital corps must obey. And it is the system which Dr. Seaman is attacking and Dr. Stokes is defending under the misapprehension that the dignity of the "department" is at stake and that the medical officer is an exclusive and infallible being.

The JOURNAL does not pretend, in the absence of complete official figures, to say what the proportion of deaths from wounds to deaths from disease has been either in the Spanish war or the Philippine insurrection; but there is evidence enough herewith, of an official character, to make it apparent that if there has been any juggling of statistics Dr. Seaman is not the sleight-of-hand performer.

Officially, here are the figures of losses during the Spanish war campaign, but only for the year 1898:

| | Deaths from Wounds | Deaths from Disease |
|---|---|---|
| Philippine Islands | 17 | 203 |
| Porto Rico | 3 | 262 |
| Cuba | 273 | 567 |
| United States | 0 | 2,649 |
| Totals, | 293 | 3,681 |

Thus, of a total of 3,974 deaths 293 were by wounds, or 1 from bullets to 13.5+ from disease, instead of the proportion of "270 to 400" or "one to one and one-half" of Dr. Stokes's paper.

It was fortunate in a way that Dr. Stokes attacked Dr. Seaman because it has served to focus attention on the latter's argument and show the value of his observations. It is quite likely, too, that when Congress gets down to it, there will be some action taken toward an abolition of the disgraceful system under which the medical corps of the army is at present laboring, and an appropriate numerical increase made in the corps. When this is done the enlisted man will be properly cared for professionally and the surgeon-general and his corps will be in a position to intelligently combat the "silent foe" which was so signally and successfully routed by the Japanese.

In this issue the JOURNAL publishes the paper read by Dr. Seaman at the Detroit meeting. It is, aside from the medical questions affecting the medical department of our army, of great interest, showing as it does the methods of the Japanese surgeons.

